# Ophthalmic artery flow direction change predicts recurrence of ischemic stroke after carotid stenting: a longitudinal observational study

**DOI:** 10.1186/s40001-022-00965-9

**Published:** 2023-01-02

**Authors:** Hui-Yi Yang, Ie-Bin Lian, Shih-Chun Wang, Ta-Tsung Lin, Yang-Hao Ou, Chi-Kuang Liu, Chih-Ming Lin

**Affiliations:** 1 Department of Mathematics, National Changhua University of Education, Changhua City, Taiwan; 2 Institute of Public Health, National Yang Ming Chiao Tung University, Taipei City, Taiwan; 3 Graduate Institute of Statistics and Information Science, National Changhua University of Education, Changhua City, Taiwan; 4 Department of Medicine Imaging, Changhua Christian Hospital, Changhua City, Taiwan; 5 Vascular and Genomic Research Center, Changhua Christian Hospital, Changhua City, Taiwan; 6 Department of Neurology, Changhua Christian Hospital, 135 Nanhsiao Street Changhua City 50006, Changhua City, Taiwan; 7 Department of Medical Imaging, Changhua Christian Hospital, Changhua, Taiwan; 8 Department of Post-Baccalaureate Medicine, College of Medicine, National Chung Hsing University, Taichung City, Taiwan; 9 Department of Social Work and Child Welfare, Providence University, Taichung City, Taiwan

**Keywords:** Carotid stent, Change of ophthalmic artery flow, Ischemic stroke, Carotid duplex, Magnetic resonance angiography, Middle cerebral artery

## Abstract

**Background and purpose:**

The implantation of carotid artery stents prevents recurrent ischemic stroke in patients with carotid stenosis. This study aimed to investigate associations between change of ophthalmic artery flow (COAF) post carotid stenting and recurrent ischemic stroke, as well as the link toward the anterior and posterior circulations and patients’ prognosis after carotid stenting.

**Methods:**

This retrospective, longitudinal cohort study recruited 87 left side carotid stenosed ischemic stroke patients undergoing left side carotid stenting between year of 2009 and 2013, and patients were followed up to 9 years after carotid procedures. Clinical data were derived from medical records. The primary outcome was stroke recurrence. Predictive factors were stenosis  > 50% in one intracranial artery and ROAF. Kaplan–Meier and Cox regression analyses were used to identify risk factors associated with stroke recurrence.

**Results:**

Among 87 included patients undergone left side carotid stent treatment, 44 had stroke recurrence within 3 years after carotid stenting. The recurrence group had significantly greater proportions of COAF after stenting (*p* = 0.001), and middle cerebral artery (MCA) and basilar artery or vertebral artery (BA/VA) stenosis > 50% (all *p* < 0.001) than the no-recurrence group. Survival was significantly shorter in patients with COAF than in those without (*p* < 0.01). Regression analysis showed that COAF was associated with stroke recurrence (HR: 3.638, 95% CI 1.54–8.62, *p* = 0.003). The recurrence rate was highest in patients with bilateral MCA stenosis  > 50% (100%), followed by left MCA stenosis  > 50% plus BA/VA stenosis  > 50% (83.33%) or COAF (82.14%). Patients with bilateral MCA stenosis  < 50% had no recurrence within 3-year follow-up.

**Conclusions:**

Prognosis after carotid stenting is poorer for patients with MCA stenosis  > 50%, BA/VA stenosis  > 50% and/or COAF. Carotid duplex and magnetic resonance angiography provide definitive information for prognosis prediction.

**Supplementary Information:**

The online version contains supplementary material available at 10.1186/s40001-022-00965-9.

## Introduction

Intracranial atherosclerosis is the main pathogenic feature of ischemic stroke worldwide. The internal carotid artery supplies the anterior circulation, which contains the anterior and middle cerebral arteries (MCA), of which the latter is noted for its involvement in acute stroke. Studies have shown that patients with carotid stenosis are at high risk for ischemic stroke and recurrent ischemic stroke [1, 2]. The ophthalmic artery is the first major branch of the internal carotid artery and reversed ophthalmic artery flow is indicative of inadequate collateral circulation [3, 4]. The incidence of reversed ophthalmic artery flow is higher in patients with severe carotid stenosis and acute stroke [3–6]. Stroke patients with reversed ophthalmic artery flow are reported to have worse outcomes than those without [3, 4, 7], while patients with MCA stenosis and reversed ophthalmic artery flow have higher risk of stroke recurrence [3]. In posterior circulation stroke, the prevalence of basilar artery/vertebral artery (BA/VA) stenosis > 50% is 20–30% in patients with ischemic stroke or transient ischemic attack, and BA/VA stenosis is also associated with recurrent stroke and mortality in these patients [8–11]. However, the associations between the anterior circulation, posterior circulation and change of ophthalmic artery flow (COAF) post carotid stenting treatment and recurrent stroke require further clarification.

Carotid stenting has demonstrated significant effects in preventing stroke recurrence in patients with carotid stenosis. However, the long-term possibility of recurrent stroke is yet to be thoroughly investigated. The primary aim of the current study is to investigate whether exist any association between pre and post stenting baseline patients’ parameters, neuroimaging studies and COAF of carotid duplex with the long-term stroke occurrence. Second, we attempt to study the long-term survival possibility of both recurrent and non-recurrent stroke groups. Lastly, with combination of intracranial and extracranial cerebral vasculature information, we hope to formulate a paradigm that enables clinical decision making.

## Methods

### Study design and population

**Fig. 1:**
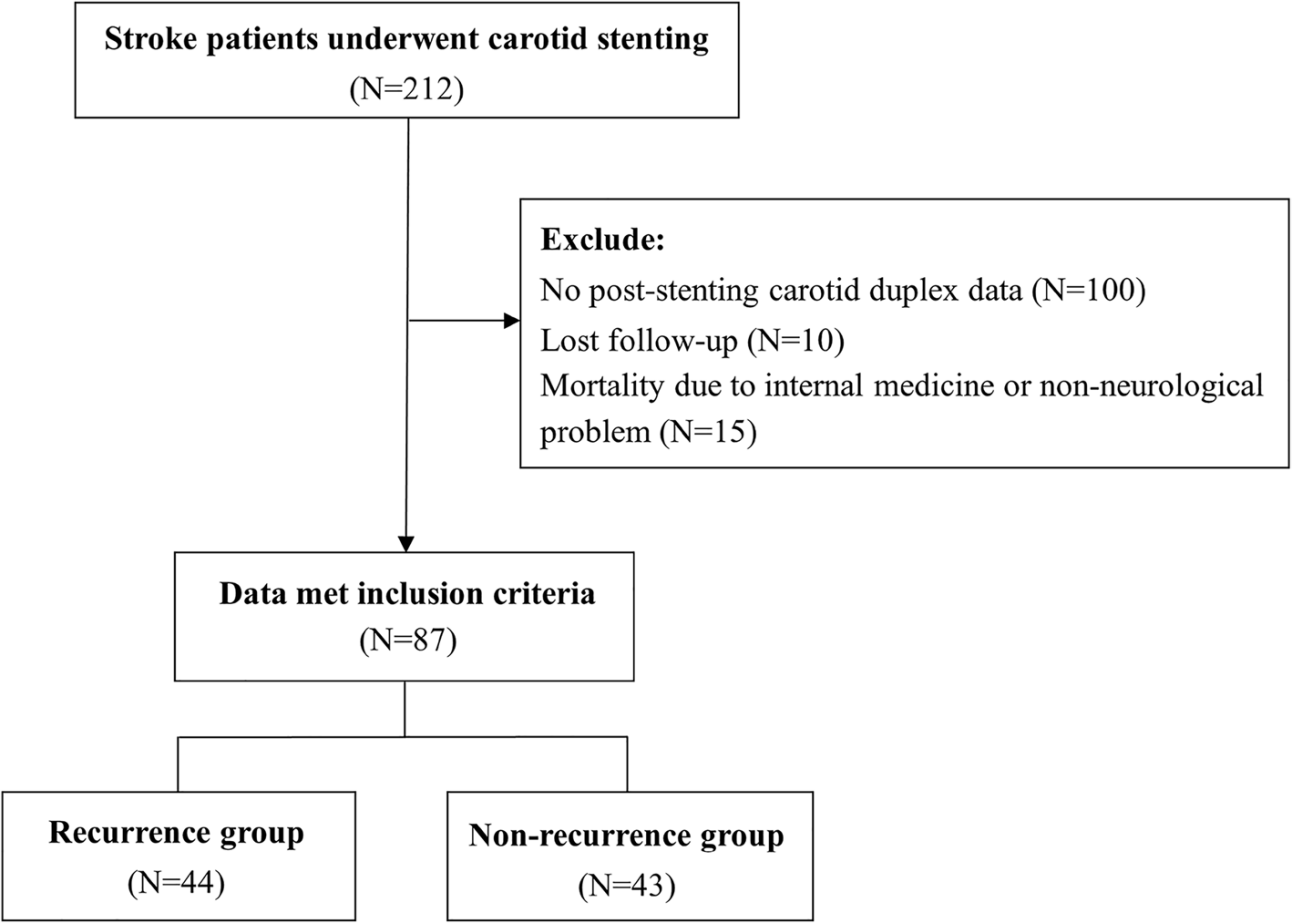
Flow chart of selection of ischemic stroke patients

The current study was a retrospective, longitudinal cohort project that recruited first time ischemic stroke patients scheduled to undergo carotid stenting at Changhua Christian Hospital between year of January/2009 and March/2013. Inclusion criteria were: (1) age ≥ 18 years; (2) No documentation of previous ischemic stroke or cerebral bleeding, tumor, or aneurysm (3) angiographic evidence of  > 70% carotid stenosis; (4) excluding of other etiologies of index stroke including cardiogenic, lacunae, or internal medicine diseases induced and no evidence of cerebral bleeding during the study period and followed for at least 12 months after stenting. Exclusion criteria were: (1) patients with cerebral hemorrhage, cerebral arteriovenous malformations, aneurysms, and bilateral moderate-to-severe carotid stenosis; (2) follow-up less than 12 months. The 87 left side carotid stenosed ischemic stroke patients were, therefore, selected for carotid stenting treatment. The study flow chart was further delineated in Fig. 1. All enrolled patients were hospitalized for medical treatment and baseline biochemistry workups. Ischemic stroke was confirmed by the diffusion weighted sequence (DWI) of magnetic resonance imaging (MRI). The carotid duplex exam was arranged and completed within 3 days upon ward admission. The second time carotid duplex examination was arranged during the neurological outpatient clinic settings and completed within 3 months after the carotid stent insertion. The diagnostic digital subtraction angiography (DSA) was arranged during hospitalization to gauge the degree of the carotid stenosis. The patients were stented within 2 weeks after the index episode (stroke event). Extracranial carotid ultrasound, magnetic resonance angiography (MRA), and CT angiography/perfusion (CTA/P) scanning were performed simultaneously before carotid stenting. All required information was obtained from medical records. The included stroke patients were followed by stroke case manager, after the neurological ward discharge, up to 9 years, and having regular outpatient clinic visiting.

### Ethical considerations

The protocol for this study was approved by the Institutional Review Board (IRB) of Changhua Christian Hospital (# 211210). Signed informed consent of participants was waived by the IRB due to the retrospective nature of the study and decoding between the original data sets and patients’ personal information.

### Carotid duplex examination

The cervical carotid artery was examined using a Philips iE33 7-MHz linear transducer (Philips Inc., Amsterdam, Netherlands) as described previously [8]. Patients tilted their heads slightly contralaterally, and the transducer was placed on their necks. Cross-sectional B-mode scanning and longitudinal screening were performed to identify intraluminal plaque and degree of stenosis, respectively. Plaque was classified into subtypes 1, 2, 3, or 4 based on the International Classification System, as previously described [12]. These parameters included peak systolic velocity (PSV), end diastolic velocity (EDV), and resistance index (RI) (PSV—EDV/PSV) of the bilateral common carotid artery, internal carotid artery (ICA), external carotid artery (ECA), and ophthalmic artery (OA) as well as COAF. Forward flow was defined as blood flow directed away from the stenotic ipsilateral carotid artery and reverse flow was defined as blood flow into the carotid artery [3, 4]. The first and second time of ophthalmic artery flow direction were compared and well-documented in the medical chart. In the current study change of flow direction was labeled as + positive, whereas − as negative. The degree of carotid stenosis was calculated according to the method used in the European Carotid Surgery Trial [13–15].

### Computed tomography angiography/perfusion scan (CTA/P imaging)

CTA examinations were performed using a second-generation dual-source CT scanner (SOMATOM Definition Flash, Siemens Healthcare, Forchheim, Germany). Perfusion data sets were postprocessed using a Siemens Multimodality Workplace Workstation (Siemens Medical, Erlangen, Germany), which calculated mean transit time (MTT), cerebral blood volume (CBV), cerebral blood flow (CBF), and time to peak (TTP). The arterial input and venous outflow curves were analyzed to ensure data set completeness. The CTP parameters are defined as follows: (1) dMTT: ipsilateral MTT—contralateral MTT. (2) MTT ratio: ipsilateral MTT/contralateral MTT. (3) MTT index: (ipsilateral MTT—contralateral MTT)/contralateral MTT. (4) dCBV: ipsilateral CBV—contralateral CBV. (5) CBV ratio: ipsilateral CBV/contralateral CBV. (6) CBV index: (ipsilateral CBV—contralateral CBV)/contralateral CBV. (7) dCBF: ipsilateral CBF—contralateral CBF. (8) CBF ratio: ipsilateral CBF/contralateral CBF. (9) CBF index: (ipsilateral CBF—contralateral CBF)/contralateral CBF. (10) dTTP: ipsilateral TTP—contralateral TTP. (11) TTP ratio: ipsilateral TTP/contralateral TTP. (12) TTP index: (ipsilateral TTP—contralateral TTP)/contralateral TTP. CTA/P imaging was performed using a second-generation dual source CT scanner (SOMATOM Definition Flash, Siemens Healthcare, Forchheim, Germany).

### Magnetic resonance imaging/angiography (MRI/A)

MRI/A was performed using a 3 T- (MagnetomVerio, Siemens Healthcare, Malvern, PA, USA) or 1.5-T imager (MagnetomAera, Siemens Healthcare) with a cervical coil.

### Angiography and carotid stenting procedure

All procedures were conducted by a neuro-interventional team in a specialized angiography clinic at the Changhua Christian Hospital, Taiwan and conducted by Dr Chi Kuang Liu. The indications for carotid stenting were the findings of internal carotid artery stenosis, with the location of suitable accessibility for the procedure to be carried out. Under general endotracheal anesthesia, one 9F femoral sheath was inserted through the femoral artery and then a Neuron Max 088 catheter (Penumbra Inc., Alameda, CA, USA) with a coaxial JB2 catheter (Cook Medical Inc., Bloomington, IN, USA) was advanced to the common carotid artery or internal carotid artery. A diagnostic cerebral angiogram was performed to confirm the location and extension of the blood clot. Next, one 8Ffemoral sheath was inserted and one 6F NeuroMax long sheath was advanced up to the stenotic site the carotid stent was inserted. The patients were sent back to intensive care unit for close blood pressure and vital signs observations post carotid stenting.

### Outcome assessment modalities

Outcome measurement parameters included the NIHSS score, the mRS, and the Barthel index, and were reevaluated at 3, 6 months, 12 months and up to 9 years upon regular neurological outpatient clinic followed ups. For each assessment, recordings were documented by both the case manager and the clinician in charge, to avoid any discrepancies. If the two recordings were too large, a third party, usually another neurologist or neuroradiologist, was invited to confirm the measures.

### Statistical analysis

All statistical analyses were performed using various packages in R software (Version 4.1.3, https://www.r-project.org/). First, all patients were classified into two groups based on whether stroke has recurred or not. Differences in characteristics between the two groups were determined by Student’s t test and are presented as means with standard deviation (mean ± SD) and proportions. The Kaplan–Meier test was used to estimate the survival probability at a given time and to observe whether changes in ophthalmic artery flow direction affects survival time up to 9 years of longitudinal observation period. The log-rank test was used to assess the significance of between-group differences. The Cox proportional hazard model was utilized to construct the recurrent stroke prediction model for exploring associations between potential risk factors and the risk of stroke recurrence. The area under the receiving operating characteristic (ROC) curve was used to measure predictive power. The Friedman test was performed to investigate the relationship between percentage of stroke recurrence and MCA stenosis or related MRA-intracranial posterior circulation artery stenosis. Decision tree is a supervisory method that allows clinicians to find the best variables impacting the targeted outcome. In the current study we utilize Classification And Regression Tree (CART) to analyzing the categorical and continuous variables, finding the minimum Gini index. The advantage of CART is that it is a binary tree analysis with Gini index as its’ analytical foundation. Gini index is also named as Gini impurity, of which the lower of the value, the better it presents the clinical outcome.

The mathematical formula of Gini:

$$\mathrm{Gini}=1-{\sum }_{i=1}^{c}{{p}_{i}}^{2}$$

A decision tree was performed to evaluate the interrelationships between middle cerebral artery stenosis, magnetic resonance arteriography intracranial artery stenosis, COAF, and stroke recurrence rate, which may help to predict risk of stroke recurrence.

## Results

The patients were recruited and enrollment process is delineated in Fig. 1. Among the 87 left side carotid stenosed ischemic stroke patients, 44 had stroke recurrence within 3 years after stenting and 43 had no recurrence within 3 years follow-up. Table 1 shows patients’ baseline demographic and clinical characteristics. No significant differences were observed between the two groups in demographics, clinical characteristics, medical history, modified Rankin scale (mRS) and Barthel index, and doppler ultrasonography of ophthalmic artery flow before stenting. The recurrence group displayed a significantly higher proportion of flow direction changes in ophthalmic artery of carotid duplex examination after stenting than the non-recurrence group (79.55% vs. 46.51%, *p* = 0.001). The proportions of intracranial artery stenosis obtained from magnetic resonance arteriography, including unilateral left side middle cerebral artery, and basilar/vertebral artery stenosis, were also significantly higher in the recurrence group than in the non-recurrence group (all *p* < 0.001). After stenting, 20.93% (9/43) of patients in the non-recurrence group and 31.82% (14/44) of patients in the recurrence group had improved mRS scores (Additional file 1: Figure S1).

**Table 1:** Demographic and clinical characteristics of ischemic stroke patients with and without recurrent strokes

	Recurrent stroke	No recurrent stroke	*p* value
Number of subjects (%)	44 (50.57)	43 (49.43)	
Age, mean, years	71.73 ± 10.10	69.72 ± 10.33	0.362
Gender (M,F)	39: 5	35: 8	0.349
BMI ± SD, mean, kg/m^2^	23.03 ± 3.07	23.32 ± 3.31	0.672
SBP ± SD, mean, mmHg	142.84 ± 23.61	137.49 ± 15.86	0.217
DBP ± SD, mean, mmHg	81.91 ± 14.32	75.09 ± 13.91	0.027
Admission mRS, mean ± SD	1.98 ± 1.39	2.21 ± 1.30	0.424
Admission Barthel index, mean ± SD	74.89 ± 30.16	78.72 ± 28.75	0.547
Smoking, *n* (%)	8 (18.18)	13 (30.23)	0.193
Education (< 12: ≥ 12)	25: 19	26: 17	0.734
Medical history, *n* (%)			
HTN	31 (70.45)	31 (72.09)	0.868
DM	21 (47.73)	13 (30.23)	0.097
CVA	17 (38.64)	15 (34.88)	0.721
CAD	7 (15.91)	11 (25.58)	0.271
Liver disease	4 (9.09)	3 (6.98)	0.721
CKD	9 (20.45)	5 (11.63)	0.268
Gout	3 (6.82)	3 (6.98)	0.977
OA, mean ± SD			
Right PSV	32.13 ± 15.28	32.33 ± 15.38	0.951
Right EDV	5.70 ± 3.71	6.07 ± 5.62	0.718
Left PSV	7.81 ± 15.71	7.42 ± 8.52	0.886
Left EDV	67.24 ± 37.72	63.14 ± 39.39	0.621
Reverse OA flow, *n* (%)			
Right	21 (47.73)	25 (58.14)	0.336
Left	26 (59.09)	19 (44.19)	0.168
Ophthalmic flow direction change*, *n* (%)	35 (79.55)	20 (46.51)	0.001
Intracranial artery stenosis, *n* (%)			
MCA right > 50% stenosis	30 (68.18)	0 (0)	< 0.001
MCA left > 50% stenosis	44 (100)	6 (13.95)	< 0.001
MRA BA or VA > 50% stenosis	28 (63.64)	11 (25.58)	< 0.001

*M* male, *F* female, *BMI* body mass index, *SBP* systolic blood pressure, *DBP* diastolic blood pressure, *mRS* modified Rankin scale, *HTN* hypertension, *DM* diabetes mellitus, *CVA* cerebrovascular accident, *CAD* coronary heart disease, *CKD* chronic kidney disease, *OA* ophthalmic artery, *PSV* peak systolic velocity, *EDV* end-diastolic velocity, *MCA* Middle cerebral artery, *MRA* Magnetic Resonance Angiography, *BA* basilar artery, *VA* Vertebral artery

^*^Ophthalmic artery blood flow direction changed on the left

**Fig. 2:**
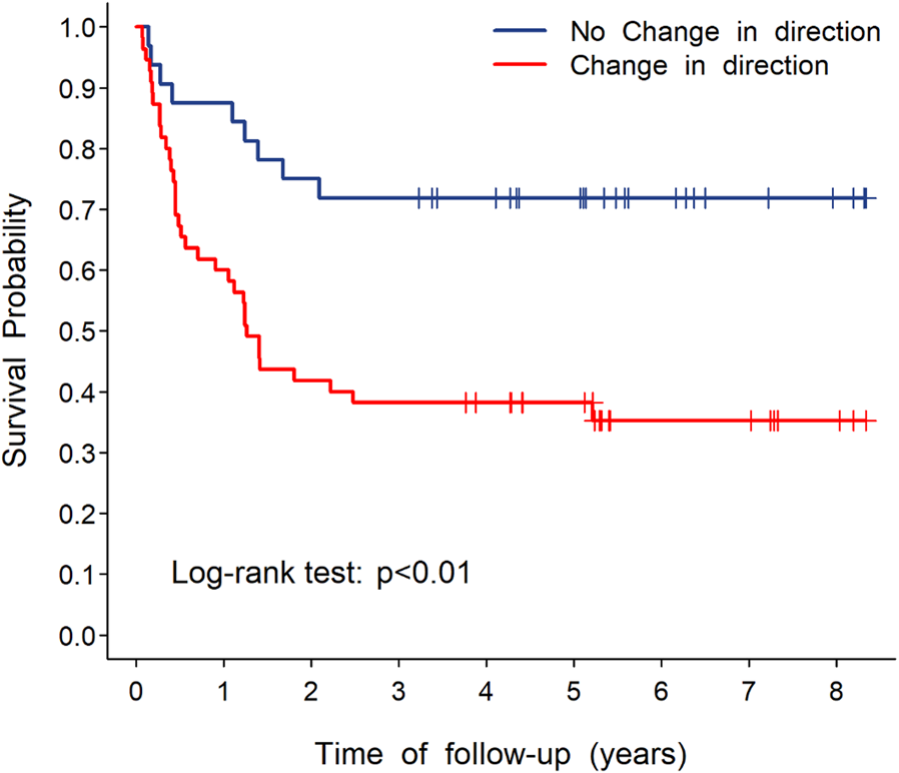
Kaplan–Meier plot of survival curve showing status of ophthalmic artery blood flow direction change after carotid stenting

Further analysis of flow direction changes in ophthalmic artery of carotid duplex examination by Kaplan–Meier plot and Cox regression showed that survival was significantly poorer in patients with flow direction changes than in those without during follow-up of more than 9 years (*p* < 0.01, Fig. 2). By selecting significant factors from univariate analysis, the following Cox regression model was obtained for log-ratio of hazard rates:

$$\text{ln}\frac{h}{{h}_{0}}= 0.0192\ ({\text{Left ICA PI}}) + 0.0186\ ({\text{Right IMT}}) + 1.2913 \ ({\text{Change of ophthalmic blood flow direction on the left}}) + 0.0166\ ({\text{SBP}}) + 0.5930\ ({\text{DM history}}) - 0.5436\ ({\text{Discharge mRS}}) - 0.0157\ ({\text{Admission Barthel index}})$$

**Fig. 3:**
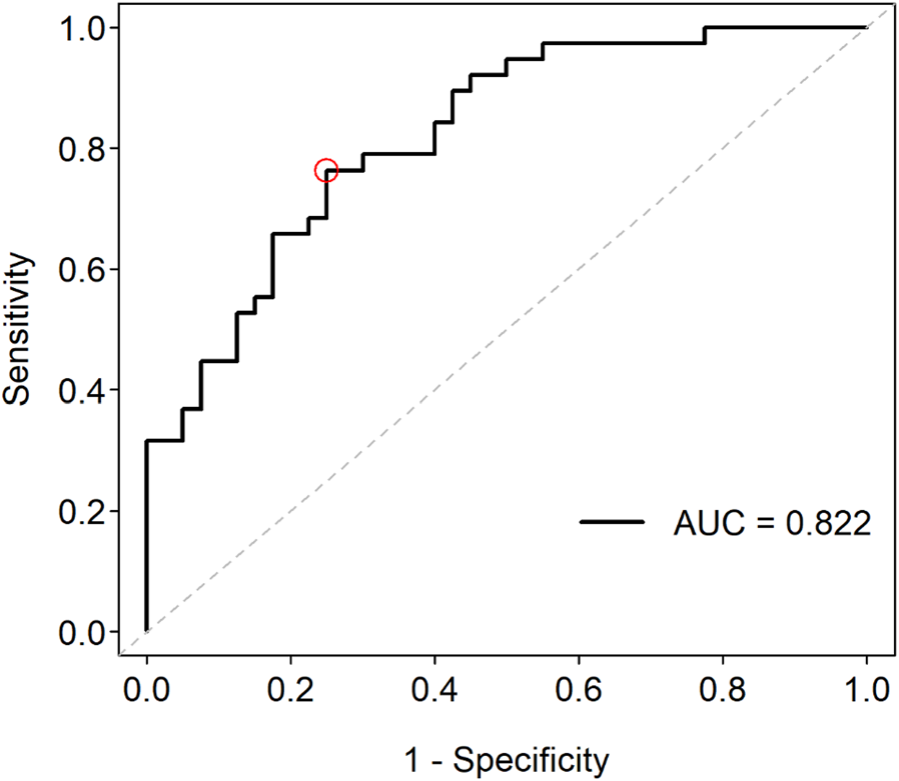
Receiver operating characteristic (ROC) curve of recurrence within 3 years after stenting, with Youden’s optimal cut-point circled in red

Wald’s tests of factors from the Cox model are listed in Table 2, which shows that after adjusting for left side internal carotid artery pulsatility index (ICA PI), right side intima media thickness (IMT), systolic blood pressure (SBP), diabetes mellitus (DM) type 2 history, and neurologic disability indices (including followed up mRS and admission Barthel Index scores), changes of flow direction of ophthalmic artery on the left increase the hazard ratio of stroke recurrence by approximately fourfold. The corresponding ROC curve of stroke recurrence within 3 years after stenting is shown in Fig. 3, for which the area under the curve was 0.822. Using the maximal Youden index, the optimal cutoff point (circled in red) of the ROC curve in Fig. 2 was selected, with log hazard ratio 1.16 as the cutoff value.

**Table 2:** Cox proportional hazards model for stroke recurrence

	HR	95% CI		*p* value
Left ICA PI	1.019	1.008	1.031	0.001
Right IMT	1.019	1.002	1.036	0.029
Change of ophthalmic blood flow direction on the left (1: + ; 0:−)	3.638	1.536	8.615	0.003
SBP	1.017	1.000	1.033	0.046
DM history	1.809	0.931	3.516	0.080
Discharge mRS	0.581	0.417	0.808	0.001
Admission Barthel index	0.984	0.972	0.997	0.016

*HR* hazard ratio, *CI* confidence interval, *ICA* internal carotid artery, *IMT* Intima Media Thickness, *PI* Pulsatility index, *SBP* systolic blood pressure, *DM* diabetes mellitus, *mRS* modified Rankin scale, *Left ICA PI* ratio change in pre- and post-stenting of pulsatility index of internal carotid artery on the left, *Right IMT* change ratio in pre- and post-stenting of intima media thickness on the right

Therefore, by plugging in the values of left ICA PI, right IMT, change in ophthalmic artery flow direction (yes as 1, no as 0), SBP, DM history (yes as 1, no as 0), mRS at discharge, and Barthel index at admission from a patient into the above linear combination, the log hazard ratio can be obtained and compared with 1.16. Patients with values  > 1.16 were identified as having higher risk of stroke recurrence.

Table 3 shows the percentages of stroke recurrence in various anterior or posterior basal artery vasculature of magnetic resonance arteriography (MRA)-intracranial artery stenosis categories. The nonparametric Friedman test indicated that stenosis in both sides and left-side stenosis had significant effects on stroke recurrence (*p* < 0.001). Right-side stenosis alone was not found in any patient. BA or VA stenosis also had significant effects on stroke recurrence (*p* < 0.01).

**Table 3:** Percentages of stroke recurrence in each intracranial major artery stenosis groups

	Recurrent stroke		No recurrent stroke		*p* value
Anterior circulation (MCA)					
Left and right > 50% stenosis	100%	(30/30)	0%	(0/30)	< 0.001
Only left > 50% stenosis	70%	(14/20)	30%	(6/20)	< 0.001
Only right > 50% stenosis	–		–		
Left and right < 50% stenosis	0%	(0/37)	100%	(37/37)	< 0.001
Posterior circulation					
BA or VA > 50% stenosis	71.79%	(28/39)	28.21%	(11/39)	< 0.01
BA or VA < 50% stenosis	33.33%	(16/48)	66.67%	(32/48)	< 0.01

*MCA* Middle cerebral artery, *MRA* Magnetic Resonance Angiography, *BA* basilar artery, *VA* Vertebral artery

**Fig. 4:**
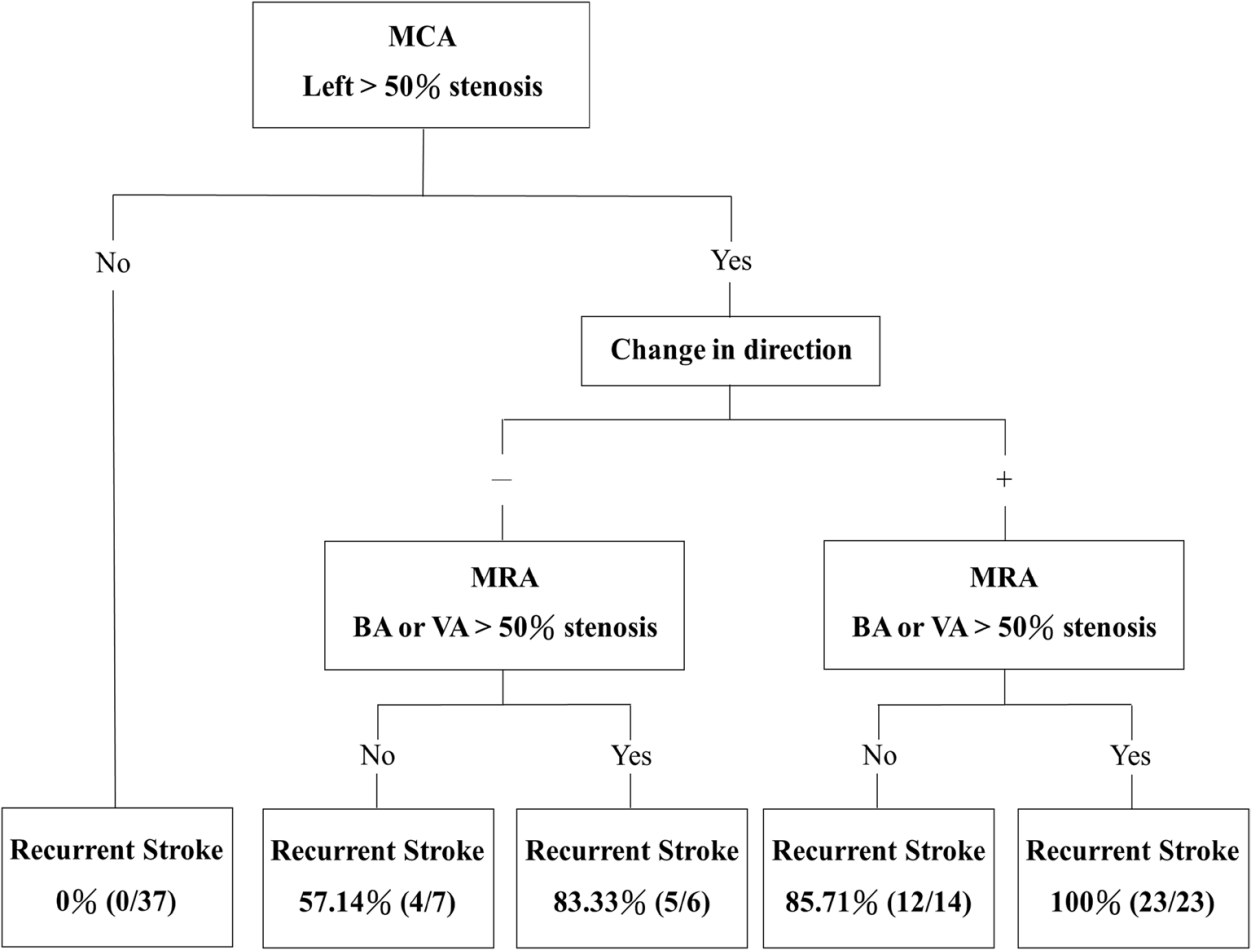
Percentages of recurrent stroke in each group categorized by MCA left stenosis, MRA BA or VA stenosis, and ophthalmic artery blood flow direction change *Note:* change in direction, ophthalmic blood flow direction changed on the left. *MCA* Middle cerebral artery, *MRA* Magnetic Resonance Angiography, *BA* basilar artery, *VA* Vertebral artery

Percentages of stroke recurrence of anterior or posterior cerebral vasculature versus ophthalmic artery flow change post carotid stent treatment are summarized in Table 4. Percentages were obtained by combining the information presented from ophthalmic artery blood flow direction change from carotid ultrasonography and MRA-based intracranial artery stenosis. Patients with  > 50% MCA stenosis on both left and right sides had the highest risk (100%), followed by those with only left-side MCA stenosis  > 50% (83%) and those with change of flow direction on the left along with BA or VA stenosis  > 50% (82%). Those who had MCA stenosis  < 50% on both sides had minimal risk (0%). The decision tree in Fig. 4 demonstrates how the information from adjunct information of both carotid ultrasonography, and in combination of anterior and posterior circulation stenoses of magnetic resonance arteriography (MRA) can be utilized and interplayed to generate the stroke recurrence prediction. It showcases the highest impact based on the decision tree is symptomatic side of intracranial middle cerebral artery stenosis (left side MCA in our study), followed by, COAF, and posterior circulation of BA/and or VA stenosis.

**Table 4:** Percentages of stroke recurrence of anterior or posterior circulation of intracranial vasculature versus ophthalmic artery flow change

MCA	Blood flow direction change*	Recurrent stroke	
Left and right > 50% stenosis	+	100%	(25/25)
	−	100%	(5/5)
Only left > 50% stenosis	+	83.33%	(10/12)
	−	50%	(4/8)
Left and right < 50% stenosis	+	0%	(0/18)
	−	0%	(0/19)
BA or VA > 50% stenosis			
Yes	+	82.14%	(23/28)
	−	45.45%	(5/11)
No	+	44.44%	(12/27)
	−	19.05%	(4/21)

*MCA* Middle cerebral artery, *MRA* Magnetic Resonance Angiography, *BA* basilar artery, *VA* Vertebral artery

^*^Ophthalmic artery flow change on the left

## Discussion

Results of the present study showed that pre-stenting parameters such as severe MCA or BA/VA stenosis and COAF are associated with stroke recurrence after carotid stenting. Stroke recurrence rates were highest in patients with severe (over 50%) bilateral MCA stenosis, followed by severe left MCA stenosis plus severe BA/VA stenosis or COAF. Survival was also significantly shorter in patients with COAF than in those without COAF.

After left carotid artery stenosis is corrected by carotid stenting, factors for recurrent ischemic stroke include the severity of stenosis in the intracranial arteries. Hence, pre-stenting parameters, including MCA and BA/VA stenoses and COAF help predict prognosis after stenting. Considering the circle of Willis, MCA is the dominant blood supply in the anterior circulation, illustrated by results showing that when MCA stenosis  < 50%, the risk for stroke recurrence is zero regardless of whether VA/BA stenosis  > 50% or not. Previous studies have also indicated that the enhanced plaque burden of MCA in MRI scans is an independent risk factor for recurrent stroke [16, 17]. In particular, significant differences were found in changes in stenosis levels and plaque enhancement between patients with/without stroke recurrence, and the changes showed correlations between stenosis and plaque enhancement, as well as with subsequent recurrent stroke [16]. Previous studies have also reported that reversal of ophthalmic artery before carotid stenting is associated with poorer prognosis [3, 4, 7]. However, high risk for recurrent ischemic stroke in patients with carotid stenosis post stenting phase is yet to be investigated. In patients with severe internal carotid stenosis or occlusion, reversal of ophthalmic artery before carotid stenting indicates an insufficient collateral circulation that may stem from intracranial hemostatic compromise [3, 4]. BA/VA is a part of the posterior circulation, and blood supply from other arteries partially supports the territory when BA/VA stenosis  < 50%. Therefore, the risk for recurrent stroke rises significantly when either BA or VA stenosis  > 50% or COAF is present under MCA stenosis  > 50%, because the blood flow to the territory distal from the internal carotid artery is largely limited.

Yang et al. [18] reported that about 1/3 Chinese patients with ischemic stroke have at least one artery stenosis  ≥ 50%, while intracranial arteries and anterior circulation arteries were susceptible to stenosis [18]. Given that tandem stenosis is frequent in Asian populations with ischemic stroke, COAF is likely to represent a concomitant stenosis of other intracranial arteries in the circle of Willis. COAF combined with other clinical and intracranial angiographic parameters helps to predict the prognosis in patients with acute ischemic stroke [3, 4, 6, 19–21]. Our previous study found that using the resistance index of carotid ultrasound before and after stenting is especially able to replace the standard use of CT perfusion exams as an assessment tool [19]. Meanwhile, carotid duplex and MRA are useful non-invasive tools that can effectively track real-time clinical conditions to provide concrete information to predict patients’ outcomes after carotid stenting.

The stronghold of the current study is the homogeneity of collected patients’ origin, of whom are all Asian origin. The carotid duplex examination is conducted by the same neuro-sonographer, which the results of the data sets are maintained at the high quality and consistency. The follow-up period is of duration of 9 years, which allows full blown demonstration of ischemic stroke patients’ medical status after carotid stent insertion. By contrast, the present study has a few limitations. First, it has the inherent limitations of its retrospective nature. Second, the sample size was small and from a single center, so that results may not be generalizable to other populations or geographic locations. Third, the stenosis levels of other intracranial arteries were not included in the investigation. Further prospective multicenter studies with larger samples are needed to expand the study parameters and confirm the present results.

## Conclusions

Pre-stenting parameters are significantly associated with recurrent ischemic stroke. The prognosis after carotid stenting is poorer for patients with severe middle cerebral artery stenosis, severe basilar/vertebral artery stenosis and/or COAF. Extracranial images such as carotid duplex and magnetic resonance arteriography help provide definitive information by which to predict the risk for stroke recurrence in patients undergoing carotid stenting.

## Supplementary Information

**Additional file 1: Figure S1. **Change in mRS scores pre- and post-carotid artery stenting in patients with and without recurrent stroke.

## Data Availability

The data sets used and/or analyzed during the current study are available from the corresponding author on reasonable request.
