# Supplementary material for: Ophthalmic artery flow direction change predicts recurrence of ischemic stroke after carotid stenting: a longitudinal observational study
Source: Eur J Med Res. 2023 Jan 2;28:1. doi: 10.1186/s40001-022-00965-9 (PMC9806874; doi:10.1186/s40001-022-00965-9)

**Appendix**

**Additional Figure 1.** Change in mRS scores before and after carotid artery stenting in patients with and without recurrent stroke


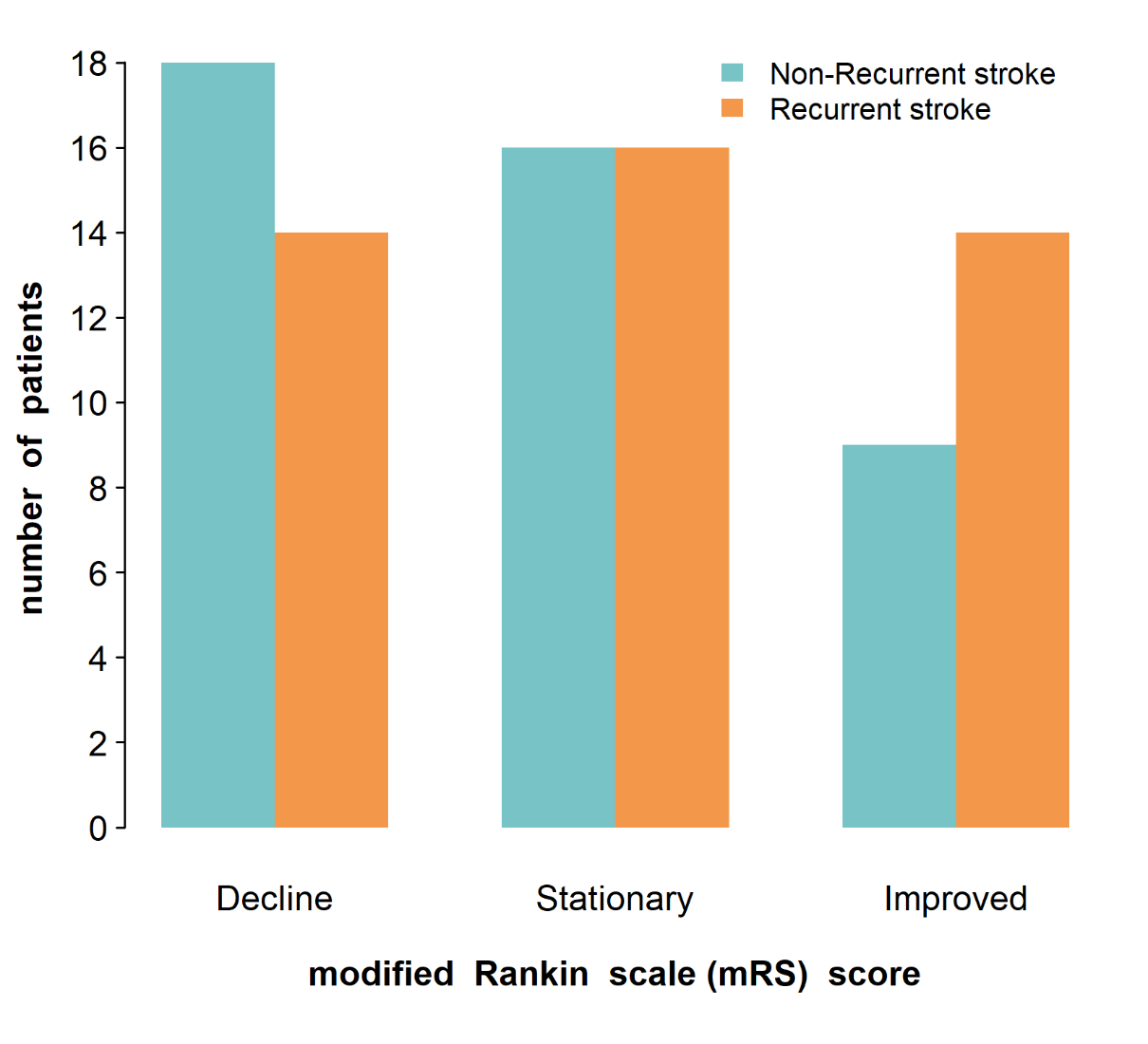

Supplement: Supplementary file 1 — Additional file 1: Figure S1. Change in mRS scores pre- and post-carotid artery stenting in patients with and without recurrent stroke. [file 40001_2022_965_MOESM1_ESM.doc]
